# Official Report of the Financial Position

**Published:** 1920-12-11

**Authors:** 


### THE LONDON HOSPITAL.

### Official Report of the Financial Position.

Salaries of the Nursing Staff.

The Committee have recently made considerable increases in the salaries of the nursing staff. To the possible criticism that such a step in the present state of the finances of the hospital is unjustifiable, the answer is that not only is the increase in scale perfectly just, since nurses have been underpaid in the past, but it is necessary on the grounds of expediency. Poor-Law infirmaries are in fact offering better salaries than are offered at the London. These increases involve an additional expenditure of £10,000 per annum, which total will increase with the annual increments of the individuals. Details of the scale of salaries have already been published in The Hospital (November 6, page 126).

The Financial Position.

The immediate difficulties of the year have been relieved by the special grant of £35,000 from King Edward's Hospital Fund and the Emergency Appeal which raised a like sum. It is recognised that a hospital without a large endowment must, to a certain extent, live from hand to mouth, but it is beyond reasonable possibility to expect that the income for next year can be increased by voluntary effort sufficiently to meet the estimated expenditure. It must not, however, be inferred from present difficulties that the Governors have any cause to regret the policy of the hospital in the past.

The income from investments, trust funds, and estate is greater now than ever before, and this despite the fact that within the last twenty-five years the hospital has been rebuilt at a cost of £727,372.

Up to 1914 the hospital paid its way, and was able to keep abreast of the improvements in building and equipment which the advances of medical and surgical science required. At the end of that year, after an expenditure of £123,334, there was a credit balance of over £9,000.

In 1919 the expenditure was £221,024, and the debit balance over £65,000. This year the expenditure will probably reach £275,000, an increase due, not to any extension of work—all proposed extensions have, in fact, been cancelled—but to the abnormal rise in prices and wages since the war.

Estimates for 1921.

Next year the estimate for expenditure is £300,000, and for income £150,000, leaving a deficit of £150,000. The Committee feel convinced that, whatever might be possible as regards a charity on a smaller scale, to increase voluntary contributions by so large a sum is impossible.

It may be said that the obvious cause is to reduce drastically the expenditure till it comes within the limits of our possible income. The Committee have gone most carefully into every possible means of cutting down expenditure, and they are convinced that to do this to a sufficient extent would mean to entirely destroy the present standard of efficiency.

The London Hospital has grown to be a national institution. In addition to being the largest hospital in England for the treatment of the sick, it is one of the largest training-schools, both for doctors and nurses, and one of the largest centres for research.

To destroy entirely the scope and efficiency of an institution whose work is national cannot be regarded as other than a disaster, and the Committee feel strongly that the time has come when, at any rate, for hospitals in the category of training, teaching, and research centres, some aid should be forthcoming from the State to supplement voluntary charity.

State or Municipal Aid.

By advocating State or municipal aid a good deal of controversy has been raised. Phrases like "the death of the Voluntary System" have been freely used, but the Committee would assure the Governors that they desire as strongly as possible the retention of the voluntary system in its essentials.

Many hospitals are already receiving State aid in some form or other. For instance, a number receive a grant for the treatment of cases of venereal disease, for children suffering from eye, skin, or throat, ear and nose complaints, for tuberculosis and for maternity and child welfare; while the finances of some hospitals are in a far better state than would otherwise be the case, owing to receiving large payments from the Government for the treatment of pensioners. Therefore, in advocating State or municipal aid, the Committee are only asking for the extension of a principle which has long since been accepted. There is no ground for supposing that the receipt of State aid in a greater degree than at present would kill voluntary charity.

Medical and surgical science, the various forms of treatment, grow daily more complex, and the Committee feel that the point has been reached when an efficient hospital service cannot be carried out by voluntary charity alone and unaided.

The Actual Present Position.

The Treasurer, Mr. W. T. Paulin, presented to the Quarterly Court the following financial statement for the current year :—

The net receipts for general purposes of the hospital have amounted to £236,457, and the hospital expects to receive by the end of the year a further £8,685, making a total of £245,142. The net payments have amounted to £243,054, and it is anticipated that payments will have to be made by the end of the year of £33,275. This makes a total of £276,329. There is, therefore, a probable deficit by the end of the year of £31,187, in spite of a special grant of £35,000 from King Edward's Hospital Fund, and another £35,000 resulting from an emergency appeal by Lord Knutsford last June.

Towards this deficit there is only any further grant that the King Edward's Fund may make, and donations from the public. The balance will have to be borrowed from the bankers, who, with the Alliance Assurance Company, hold practically all the realisable investments as security for loans already made, amounting to £66,000, and the right to borrow a further £34,000 as required.

